# Synthesis of In Situ Photoinduced Halloysite-Polypyrrole@Silver Nanocomposite for the Potential Application in Humidity Sensors

**DOI:** 10.3390/nano10071426

**Published:** 2020-07-21

**Authors:** Khouloud Jlassi, Shoaib Mallick, Hafsa Mutahir, Zubair Ahmad, Farid Touati

**Affiliations:** 1 Center for Advanced Materials (CAM), Qatar University, Doha 2713, Qatar; 2 Department of Electrical Engineering, College of Engineering, Qatar University, Doha 2713, Qatar; sm1404760@student.qu.edu.qa (S.M.); touatif@qu.edu.qa (F.T.); 3 Department of Chemical Engineering, College of Engineering, Qatar University, Doha 2713, Qatar; hm1708761@student.qu.edu.qa

**Keywords:** halloysite-polypyrrole-silver nanocomposite, humidity sensor, response and recovery times, hysteresis

## Abstract

Halloysite-polypyrrole-silver nanocomposite has been prepared via in situ photopolymerizations of pyrrole in the presence of silanized halloysite and silver nitrate as a photoinitiator. The halloysite nanoclay (HNT) was modified using the hydrogen donor silane coupling agent (DMA) in order to provide anchoring sites for the polypyrrole/silver composite (PPy@Ag). The mass loadings for both PPy and Ag have been estimated to be 21 and 26 wt%, respectively. The anchored Ag particles were found in the metallic state. The resulting PPy@Ag-modified silanized HNT has been evaluated for the potential application for impedance humidity sensors. HNT-DMA-PPy@Ag nanocomposite with different weight % of PPy@Ag (0.25 wt%, 0.5 wt%, and 1 wt%) was deposited on the pre-patterned interdigital Indium Tin Oxide (ITO) electrodes by spin coating technique. The addition of Ag nanoparticles within the nanocomposite enhances the hydrophilicity of the sensing film, which improves the sensitivity of the humidity sensors. The HNT-DMA-PPy@Ag (0.5 wt%) nanocomposite-based impedance sensors showed good sensitivity and lowered hysteresis as compared to the other ratios of the composite. The maximum calculated hysteresis loss of the HNT-DMA-PPy@Ag (0.5 wt%)-based humidity sensor is around 4.5% at 80% RH (relative humidity), and the minimum hysteresis loss estimated to be 0.05% at 20% RH levels. The response and recovery time of HNT-DMA-PPy@Ag (0.5 wt%) nanocomposite-based impedance sensors were found to be 30 and 35 s, respectively. The interesting humidity-dependent impedance properties of this novel composite make it promising in humidity sensing.

## 1. Introduction

Humidity sensors are widely used in environmental monitoring, particularly in the human comfort zones and industrial manufacturing processes [1]. A high-performance humidity sensor must have a quick response time, high sensitivity, low hysteresis, and excellent stability [2,3]. The contemporary need for high-performance humidity sensors has led to rigorous research and development (R&D) in this area during the last decade. To achieve high sensitivity, long-term stability, fast response, and recovery time, reduce hysteresis, and cost-effective of humidity sensors, various types of humidity sensing materials have been investigated, which mainly includes nanoporous materials, ceramics, and polymers. Among the polymeric materials, polypyrrole (PPy) has been broadly investigated for many applications due to its easy preparation, mechanical stability, and interesting electrical properties [4,5,6]. The electrical properties of PPy are very sensitive to the change in the relative humidity levels, which make it a very promising candidate for potential application in humidity sensors [7,8]. PPy can be prepared through several techniques including oxidative polymerization, electro-polymerization, and in situ photopolymerizations in presences of photoinitiators such as copper, gold, or silver-based metallic salts [9,10]. These metallic initiators oxidize the pyrrole monomer and consequently reduce to stable metallic nanoparticles, trapped in the polypyrrole polymer matrix [11]. In situ photopolymerization is considered as the most efficient process, because of the use of photoinitiators and monomers [12], mainly the selected monomers could be grafted in situ of the modified surface through chemical bonding reaction with high efficiency [13]. In the current study, ppy has been hybridized with the inorganic materials halloysite (HNT), which is kaolinite based naturally abundant clay mineral and possesses good chemical stability, high porosity, large surface area [14]. The unique tubular structure and high surface area of HNT improve the physicochemical properties of the HNT based nanocomposites [15]. To enhance the water vapors adsorption in HNT, the incorporation of hydrophilic materials in HNT is a well-known approach [16,17,18]. Herein we wished to investigate the propensity of HNT as low cost, bio-based micro-platforms for the immobilization of ppy/Ag metallic nanoparticles. In this regard, HNT surface pretreatment aspect is important via ppy/Ag metallic nanoparticle immobilization. A close look at the surface composition of HNT highlighted the presence of hydroxide groups at the outermost surface, a fact that is line with their hydrophobic character. We thus reasoned modifying the HNT surface with hydrogen donor silane in a similar way to silica and other reported materials [19], this will induce the formation of PPy rich surface with the presence of silver nanoparticles in metallic state, as previously reported in our group, on silica and bentonite clay [11,19]. In this way, we aimed to design new hybrid functional materials (relevant to humidity sensing) by valorizing HNT natural nanoclay. To the very best of our knowledge, such an investigation on HNT modification with hydrogen donor silane, to anchor, well-dispersed PPy/Ag through one pot and ultrafast photochemistry, has never been conducted previously. Moreover, previous studies showed that ceramic materials doped with silver nanoparticles, as proven to have a positive effect on the efficiency of humidity sensors, with the addition of an optimized concentration of silver was proven to improve the sensitivity of the sensors and reduce the response time compared to pristine [11,20]. Thus, HNT nanocomposite, doped with silver nanoparticles as well as PPy, are expected to present superior humidity sensing properties due to their ability to improve electrical properties of HNT, as well as their high water vapor molecules adsorption capabilities, through the pore openings present in PPy surface.

This study aimed to develop a fast, cost-effective, and efficient humidity sensor using the novel hydrophilic organic-inorganic hybrid materials. Here, halloysite was selected as an inorganic template; this latter was modified using hydrogen donor silane: *N,N-dimethylaminopropyltrimethoxysilane* (DMA), to design a hybrid macro photoinitiator for pyrrole/silver anchoring, through easy one post radical in situ photopolymerizations.

## 2. Materials and Methods

Materials: All the chemicals, including Pyrrole, Silver nitrate, *N, N-dimethylaminopropyltrimethoxysilane*, and the HNT clay, were purchased from Aldrich.

Silanization of halloysite: HNT was first activated with HCl solution (1mol/L) under room temperature for 72 h, then centrifuged and washed several times. HNT (5.0 g) was then mixed with DMA, a coupling agent (20 mL) in toluene (250 mL). The solution was then stirred for 72 h at 80 °C under the nitrogen atmosphere. The modified HNTs were then centrifuged, followed by washing using ethanol. Finally, the material was dried in vacuum at 50 °C for 24 h to obtain an amino-functionalized halloysite, hereafter called HNT-DMA.

Preparation of silanized halloysite/polypyrrole-silver hybrid materials: The silanized halloysite/polypyrrole-silver hybrid materials were tailored through the in situ photochemical-polymerization technique. One gram of HNT-DMA was dissolved in 100 mL of deionized water followed by the addition of the AgNO3 aqueous solution (0.84 g in 50 mL) and pyrrole (1.2 g in 40 mL corresponding to 0.5 mol/L) under stirring. Afterward, the mixture was transferred to a glass vessel, and placed 15 cm below the six lamps of the UV-reactor (Spectrolinker 1500) and irradiated at the wavelength of 365 nm for 20 min. The resulting material was centrifuged then washed several times with deionized water and ethanol to remove unreacted monomers. Then dried in vacuum at 40 °C for 48 h to obtain silanized halloysite/polypyrrole-silver hybrid materials, hereafter called HNT-DMA-PPy@Ag nanocomposite.

**Figure 1:**
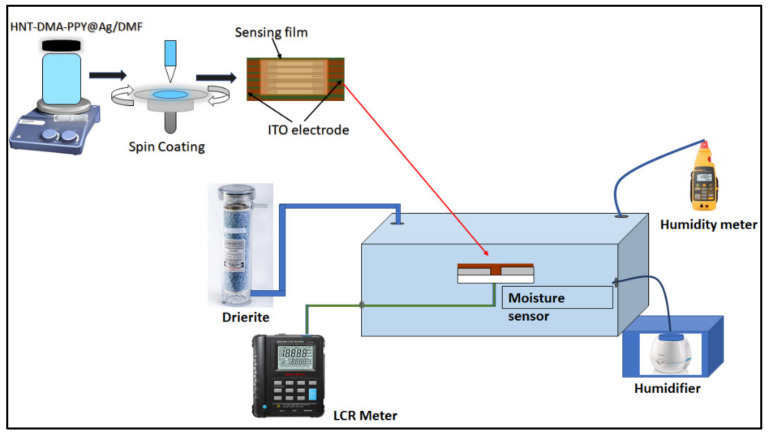
Graphical illustration of HNT-DMA-PPy@Ag based humidity sensor prepared by the spin coating method and humidity sensor setup used during this work [21].

Preparation of humidity sensor-based HNT-DMA-PPy@Ag: HNT-DMA-PPy@Ag nanocomposite with different concentrations of PPy@Ag (0.25 wt%, 0.5 wt%, and 1 wt%) prepared by dispersing in DMF. The spin coating technique has been used to deposit the HNT-DMA-PPy@Ag nanocomposite solution on the ITO/glass electrode. The interdigitated ITO electrodes (S161) were purchased from Ossila UK. First, the ITO electrode cleaning process was performed by sonicating the substrate in soap water for 10 min and placing the electrode in distilled water. After that, the ITO electrodes were sonicated in acetone and distilled water for 10 min each and then the substrate was dried with dry nitrogen gas. During the deposition of sensing film, an optimization process has been done for the rotation speed and the rotation time to form an even equilateral spread of the solution. The rotation speed and rotation time were optimized to 5000 rpm and 50 s. Figure 1 shows the graphical representation of the fabrication process of the HNT-DMA-PPy@Ag nanocomposite humidity sensor.

Characterization: The morphology of the prepared materials and films was investigated using scanning electron microscopy (SEM, FEI NOVA Nano-450, Netherlands), transmission electron microscopy (TEM, FEI, TALOS F200X, USA), and atomic force microscopy (AFM, MFP-3D, Asylum Research, USA) machine. The X-ray diffraction (XRD) measurements were performed using a PANalytical instrument (model X’PertPRO) with Co Kα (1.789 Å) radiation. Raman spectroscopy of the prepared composite was recorded using Thermo fisher scientific DXR. Raman Microscope, with a laser source of 633 nm. Energy dispersive X-ray (EDX) analysis was performed with an EDAXGENE-SIS analyzer installed on a scanning electron microscope (SEM6100 JEOL). The composition of the as-prepared HNT-DMA-PPy@Ag composites determined by elemental analysis using a Perkin-Elmer2400 CHN elemental analyzer. X-ray photoelectron spectra (XPS) were obtained using a Thermal VG ESCALAB 250 instrument fitted with a monochromatic Al Kα X-ray source. The hydrophilicity of the HNT-DMA-PPy@Ag nanocomposite film was assessed by the optical contact angle measurement (tapping the sessile drop method and using the SCA software). The electrical response of the HNT-DMA-PPy@Ag-based impedance humidity sensors was carried out in the controlled humidity chamber as reported in our previous work [21]. The electrical characterization of the HNT-DMA-PPy@Ag-based impedance sensors performed in a sealed chamber. The HNT-DMA-PPy@Ag (0.5 wt%)-based impedance sensors showed higher sensitivity and lowered hysteresis as compared to the other ratio of the nanocomposite. Figure 1 illustrates a graphical demonstration of the used humidity sensor set up.

## 3. Results and Discussion

**Figure 2:**
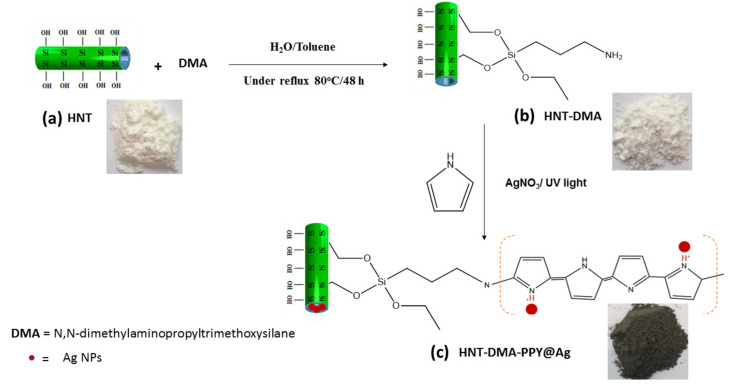
Sequential modification steps of halloysite nanoclay (HNT) by *dimethylaminopropyltrimethoxysilane* (DMA) coupling agent, followed by in-situ photopolymerization of Py. The digital photographs show HNT (**a**), silanized halloysite (**b**), and HNT-DMA-PPy@Ag (**c**).

Preparation and visual inspection of HNT-DMA-PPy@Ag: Figure 2 shows the consecutive steps for the HNT-DMA-PPy@Ag hybrid material preparation. After the acid activation, the HNT was first silanized using the DMA coupling agent. Subsequently, it was used as a macro-initiator for the pyrrole photopolymerization in the presence of AgNO_3_ as a photosensitizer [22]. In Figure 2, the digital photograph (a) shows that the pristine HNT is like a white dry powder. After grafting the DMA layer on the surface of HNT clay, it shows a sticky texture (photograph b), demonstrating the sequential modification steps of the HNT surface. Digital photo (c) displays black colored HNT after the in-situ preparation of PPy@Ag.

**Figure 3:**
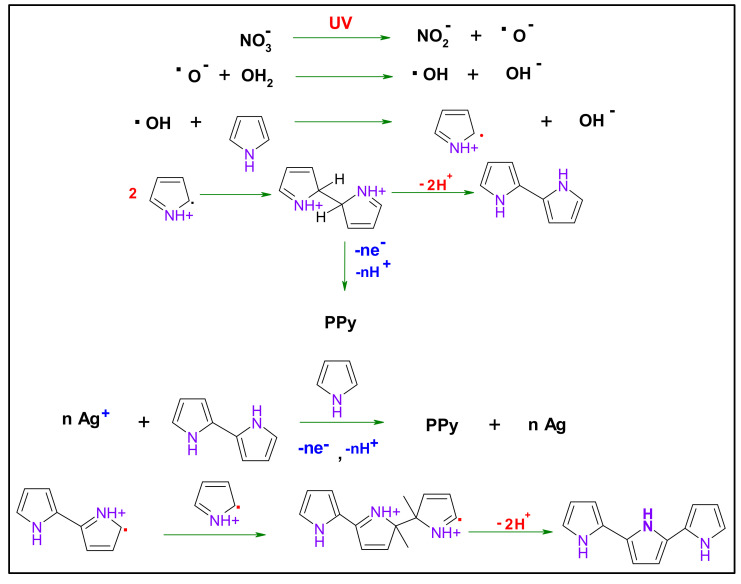
Pyrrole photopolymerization mechanism in the presence of silver nitrate photosensitizer [23].

Figure 3 demonstrates the mechanism of polypyrrole formation under UV–irradiation. In the presence of AgNO_3_ as a sensitizer, first, the Ag^+^ in the excited state strips an electron from the pyrrole monomer; this results in oxidation of pyrrole leading to (radical cations) and Ag^+^ reduction to the metallic state. Two of the present radicals may couple and led to dimerization with deprotonation, forming bi-pyrrole. This latter may be reoxidized and then coupled with other radicals. This latter may react with Py free radical, leading to PPy chain growth [23].

**Figure 4:**
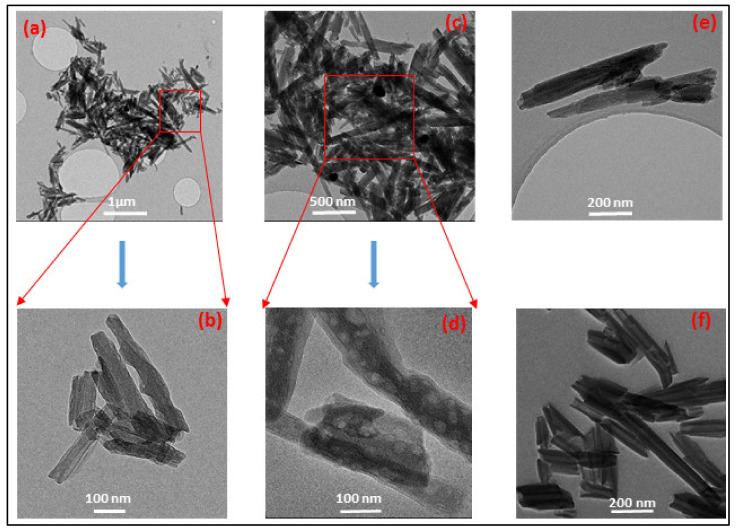
TEM images of HNT-DMA-PPy@Ag nanocomposites (**a–e**) and unmodified HNT (**f**).

Morphology and structure: Figure 4a,f show the TEM images of the PPy@Ag coated HNT and HNT, respectively. It was observed that the surface morphology of HNTs was altered as well after coating with PPy. Figure 4f exhibits the tubular structures of the nanoclay, with an average diameter of 30 nm and smooth surface morphology. Figure 4a–e, shows that after surface modification followed by in situ photopolymerizations, PPy@Ag particles are located on the HNTs’ surfaces as well as wrapped inside and at the surface of the HNT tubes [24,25], as shown in the magnified Figure 4b,d,e). The tube diameter was observed to increase up 100 nm (Figure 4e), and an increase in surface roughness of PPy-modified HNTs was observed.

**Figure 5:**
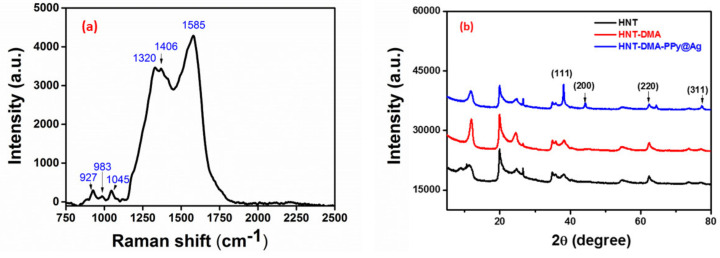
(**a**) Raman spectra of HNT-DMA-PPy@Ag, (**b**) XRD pattern of HNT, HNT-DMA, and HNT-DMA-PPy@Ag nanocomposites.

Raman spectroscopy was performed in order to characterize the structure of the synthesized nanocomposite. Figure 5a Shows the Raman spectrum of HNT-DMA-PPy@Ag at room temperature. The Raman peaks obtained for HNT-DMA-PPy@Ag come from the PPy part of the composite. The peaks, which appeared at 1585 cm^−1^, are attributed to the C=C backbone stretching bonds [26]. The peaks at 1406 and 1320 cm^−1^ are attributed to the antisymmetric C–N stretching and to the ring stretching of the PPy, respectively [27], whereas the peak located at 1045 cm^−1^ is related to the symmetrical C–H in-plane bending [28]. The peaks at 984 and 927 cm^−1^ are assigned to ring deformation vibrations [27]. These results demonstrate that HNT-DMA-PPy@Ag Raman spectra are consistent with previously reported PPy-based materials. The XRD patterns of the HNT-DMA and HNT-DMA-PPy@Ag nanocomposites are shown in Figure 5b. One can note the presence of reflection peak situated at 11.9°, corresponding to the basal distance of the HNT clay d(001), moreover the presence of diffraction peaks at 19.9°, 24.5°, 35.1°, 38.3°, 54.6°, and 62.4° which confirm that the crystalline structure of HNT clay is well maintained in all samples [29], after pretreatment and upon photopolymerization (Figure 5b) The presence of diffraction peaks at 38.2°, 44.4°, 64.4°, and 77.39° are corresponding to the (111), (200), (220), and (311) diffraction planes of Ag, respectively [30]. These patterns confirm metallic nature of Ag nanoparticles, resulted upon the photopolymerization of Py in the presence of AgNO_3_ [11], The AgNPs average size was equal 82 nm, the latter was estimated from the full width half maximum (FWHM) of the highest intensity (111) peak of Ag. However, no diffraction peak was noted for PPy polymer, most probably because of the thin layer formed upon photopolymerization and amorphous nature of the PPy [31].

The elemental composition of the final nanocomposite and pristine (Table 1) was evaluated through EDX and by CHN elemental analyzer. The values were averaged over five analyses. The PPy carbon content was estimated to be 27.1% in the final hybrid material. The mass loading of PPy to 21% and that of silver NPs to 26% in the final HNT-DMA-PPy@Ag nanocomposite. The nitrogen, carbon, and hydrogen content using CHN elemental analyzer was found to be (3.5, 40, and 6 wt%), respectively. Moreover, the calculated PPy content in HNT-DMA-PPy@Ag composites was determined from the nitrogen content of the HNT-DMA-PPy@Ag and found to be 15 wt% composites as previously reported [32].

**Figure 6:**
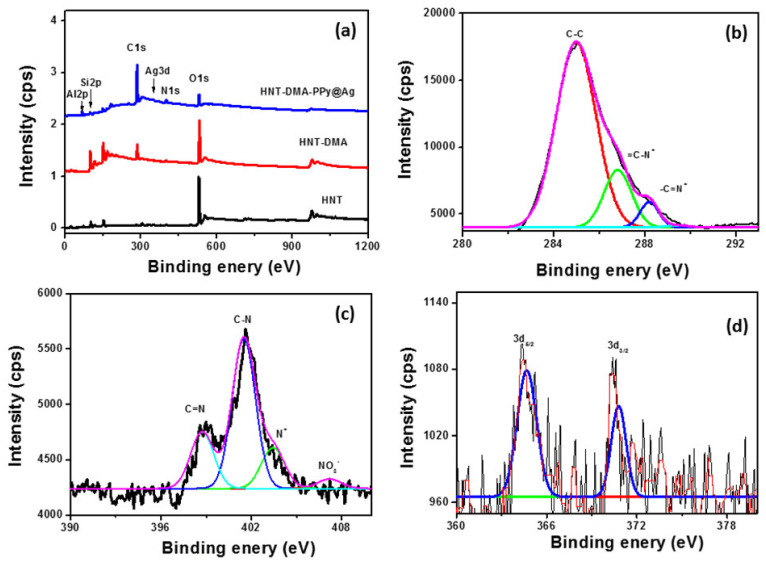
(**a**) XPS survey regions of HNT, HNT-DMA, and HNT-DMA-PPy@Ag, High resolution (**b**) C1s, (**c**) N1s, and (**d**) Ag 1d regions from modified halloysite samples.

The XPS survey spectra for the HNT, HNT-DMA, and HNT-DMA-PPy@Ag samples are presented in Figure 6. The anticipated elements in the samples are carbon, nitrogen, silicon, aluminum, sodium, silver, and oxygen. The presence of carbon and nitrogen are foreseeable from the chemical composition of HNT and HNT-DMA. However, the silver is resulting due to the PPy@Ag. Upon photopolymerization, the C1s region (Figure 6b) clearly shows an inflection point because of the addition of the beta carbon atom type from the polypyrrole. It is important to note that the peak is also tailing due to the conjugation of the polymer backbone, and which was confirmed by previously published work [31]. Concerning the N1s (Figure 6c), we can note the presence of the C=N component at ~399 eV; however, the presence of the element at high binding energy side is assigned to the positively charged nitrogen atoms at ~403 eV. It is nice to note the presence of a small feature at ~406.1 eV, which is assigned to the nitrate, acting here as dopant [11]. The N1s, C1s, and Ag3d features have very low relative intensities. This is supported by quantitative data reported in Table 2. A doublet of Ag3d was observed upon photopolymerization in the HNT-DMA-PPy@Ag sample, indicating that the success of the used process.

The surface chemical composition of HNT-DMA-PPy@Ag material and precursors was reported in Table 2. After the Silanization of HNT, a small increase was noted in carbon and nitrogen content; however, after polymerization, a greater extent of C, N, and Ag was noted while depletion of the atomic percentages for the aluminosilicate elements (Si, Al, and O). Nitrates were detected only in the presence of HNT-DMA-PPy@Ag. Indeed, the pretreatment of PPy using a silane coupling agent provides more anchoring sites for polypyrrole. Thus, HNT is no longer enough to counterbalance and neutralize the charge, hence the presence of the nitrates on the HNT-DMA-PPy@Ag surface.

**Figure 7:**
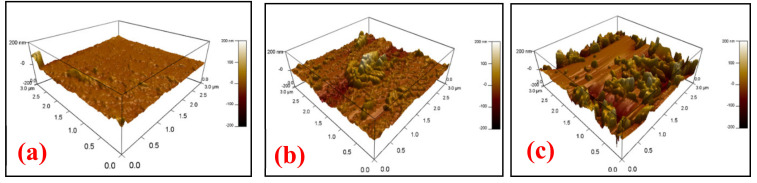
The atomic force microscopy AFM image of HNT-DMA-PPy@Ag nanocomposite film (**a**) 0.25 wt%, (**b**) 0.5, and (**c**) 1 wt% of HNT-DMA-PPy@Ag.

Morphology of the sensing film: The AFM analysis performed to study the surface roughness and morphology of the HNT-DMA-PPy@Ag nanocomposite sensing films. Figure 7a–c shows the AFM image of the HNT-DMA-PPy@Ag nanocomposites film with (0.25, 0.5, and 1 wt%) of the hybrid composite film, respectively. It can be observed that as the concentration of PPy@Ag in the nanocomposite increases the roughness of the sensing film increases as well. The developed rough structure of the composite film enhances the water molecules capture over the surface of the composite film, which will increase the sensitivity of the HNT-DMA-PPy@Ag-based humidity sensors. The AFM image (Figure 7b) of HNT-DMA-PPy@Ag (0.5 wt%) shows a relatively uniformly distributed rough surface as compared to the other samples.

The hydrophilicity of the sensing film: The hydrophilicity of the film indicates that the capacity of a sensing film towards the absorption of the water molecules. The contact angle of water drops on the surface of the sensing film measured by the sessile drop method. Table 3 shows the contact angle measurements of the HNT-DMA-PPy@Ag (0.25, 0.5, and 1 wt%)-based sensing film. The contact angle of HNT-DMA-PPy@Ag nanocomposite with 0.25 wt% of PPy@Ag is 68.2°, which shows its hydrophilic nature. The contact angle of HNT-DMA-PPy@Ag nanocomposite with 0.5 wt% of PPy@Ag is decreased to 46.6°. This decrease in contact angle shows that the addition of PPy@Ag improved the sensing film hydrophilic property. This increase in hydrophilicity enhances the water vapor absorption over the surface of the sensing film, which as a result increases the sensitivity of the sensors at lower humidity levels. With the increase in the concentration of PPy@Ag (1 wt%), the sensing film becomes highly hydrophilic, and contact angle decrease to 37°. The highly hydrophilic surface entraps more water vapors; as a result, the sensitivity of the sensing film further increased. However, in highly hydrophilic surfaces, water molecules adsorbed on the sensing film and formed a strongly H-bonding with sensing film surface that will slow the desorption process [33].

**Figure 8:**
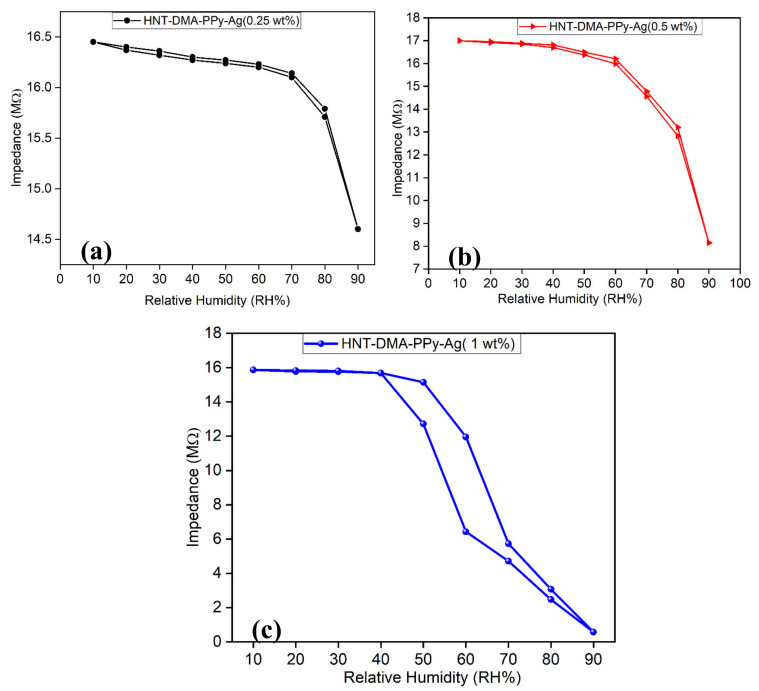
Impedance vs. relative humidity level for fabricated HNT-DMA-PPy@Ag nanocomposite humidity sensors operating temperature is 25 °C at 10 kHz(**a**) Hysteresis response of HNT-DMA-PPy@Ag (0.25 wt%) (**b**) Hysteresis response of HNT-DMA-PPy@Ag ( 0.5 wt%) (**c**) Hysteresis response of HNT-DMA-PPy@Ag (1 wt%).

Potential humidity sensor application: Photopolymerization of pyrrole in the presence of modified HNT is interesting for three main reasons: it investigates the propensity of HNT clay as a natural material with high surface area, low cost, used hereafter as bio-based micro-platforms for the in situ photopolymerizations of pyrrole. The reaction was ultrafast and mainly yielded polypyrrole with anchored silver NPs. Given the anchored PPY/Ag NPs anchored in the final HNT-DMA-PPy@Ag nanocomposite, it is possible to interrogate the performances of this HNT-DMA-PPy@Ag in humidity sensing filed. The humidity sensing response of the fabricated HNT-DMA-PPy@Ag nanocomposite impedance sensors characterized inside the controlled humidity chamber over the wide RH range (10RH–90%RH). Figure 8 shows the impedance response of the HNT-DMA-PPy@Ag nanocomposite sensors with different concentrations of PPy@Ag. The impedance of the humidity sensor decreases with an increase in humidity level. As the humidity level increases, a higher concentration of water molecules formed on the surface of the sensing film; hence more water molecules adsorbed on the sensing film, which increases the conductivity of the nanocomposite film. The humidity response of pure HNT-based humidity sensor (Figure S1 Supplementary Materials file) was measured and it shows a very low sensitivity at lower humidity levels (20–60% RH) compared to modified HNT samples. The incorporation of PPy@Ag within HNT enhances the sensitivity of the humidity sensor at a lower humidity level aswell. L. Kabir et al. [34] reported that the addition of silver nanoparticles within PPy significantly increases the conductance of the sensor, which as a result, improves the humidity sensitivity of the PPy-Ag nanocomposite-based humidity sensor. Also, the incorporation of PPy@Ag within HNT increases the dispersion of PPy@Ag, as well as the hydrophilicity of the nanocomposites sensing film, which is shown by the contact angle measurement method. This increase in hydrophilicity improves the humidity sensitivity of the sensor [35]. Figure 8(a–c) show the hysteresis response of the HNT-DMA-PPy@Ag nanocomposite-based humidity sensors prepared with different concentrations of ppy@Ag (0.25, 0.5, and 1 wt%). Hysteresis is an important parameter to analyze the sensor’s performance. The hysteresis effect can be described as the maximum difference in impedance when the sensor is exposed to change in relative humidity level from lower humidity level to higher humidity level (absorption process) and from higher humidity level to lower humidity level (desorption process). It can be observed from Figure 8a at a lower concentration of the PPy@Ag (0.25 wt%), the sensor shows lower sensitivity with respect to change in humidity level. As the concentration of PPy@Ag increase to 0.5 wt%, the sensitivity of the impedance sensor enhances due to an increase in hydrophilicity of the sensing film, which enables it to operate at lower humidity levels as well. Further increasing the concentrations of PPy@Ag (1 wt%), sensing film becomes a super hydrophilic surface, which is shown in contact angle measurement analysis (Table 3). This increase in hydrophilicity not only increases the sensitivity of the sensor, but it also slows down the desorption process of the impedance sensors while decreasing the humidity level inside the humidity chamber, which as a result increases the hysteresis loss. The HNT-DMA-PPy@Ag (0.5 wt%) nanocomposite-based impedance humidity sensors show more regular response and lower hysteresis loss as compared to the other concentrations of PPy@Ag (0.25 wt% and 1 wt%). The maximum hysteresis loss of the HNT-DMA-PPy@Ag (0.5 wt%)-based humidity sensor calculated to be 4.5% at 80% RH levels and the minimum hysteresis loss estimated to be 0.05% at 20% RH levels.

**Figure 9:**
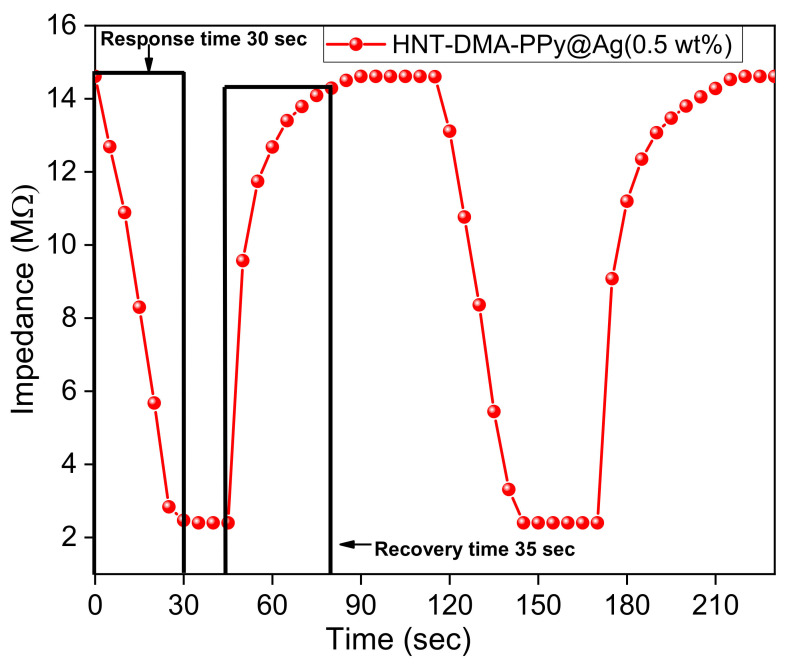
The response and recovery cycle (40–95% RH) of HNT-DMA-PPY@Ag (0.5 wt%) nanocomposite-based impedance sensor.

The response and recovery times of the impedance humidity sensors are also essential characteristics in determining the performance of a sensor. The response time of the humidity sensor defined as the time taken by the sensor to attain 90% RH (in the current study: 40–95% RH). The recovery time defined as the time that a sensor requires to reach the initial RH level (in this case, 40% RH) from 90% RH. Figure 9 shows the stable and repeatable response and recovery curve of HNT-DMA-PPy@Ag (0.5 wt%)-based impedance sensors. The response and recovery time of the HNT-DMA-PPy@Ag (0.5 wt%)-based impedance sensor evaluated to be 30 s and 35 s, respectively, at a room temperature of 25 °C. Table 4 shows the comparison of our work with previously reported PPy@Ag sensors. Our newly synthesized material has shown the significant improvements to the response and recovery times as compared to the previous study on the PPy systems.

## 4. Conclusions

Halloysite-polypyrrole–silver nanocomposite was successfully prepared via in situ photopolymerizations of pyrrole in the presence of silanized halloysite and silver nitrate as a photo-initiator. We have modified the halloysite nanoclay (HNT) using hydrogen donor silane and coupling agent (DMA) to provide anchoring sites for the polypyrrole/silver composite (PPy@Ag). Both PPy and Ag mass loading were estimated to 21 and 26 wt%, respectively. The anchored Ag particles were found in the metallic state, and the resulting HNT-DMA-PPy@Ag nanocomposite was black because of the PPy-rich modified surface. The resulting PPy@Ag-modified silanized HNT was finally evaluated for impedance humidity sensing using (0.25, 0.5, and 1 wt%) HNT-DMA-PPy@Ag nanocomposites. We achieved improved hysteresis and stable response with (0.5 wt%) HNT-DMA-PPy@Ag of the added nanocomposite. The HNT-DMA-PPy@Ag (0.5 wt%) nanocomposite-based impedance sensors showed high sensitivity, stable response, and low hysteresis as compared to other ratios of PPy@Ag (0.25, 1 wt%). The response and recovery time of HNT-DMA-PPy@Ag (0.5 wt%) impedance sensors calculated to be 30 s and 35 s, respectively. From the above, we highlight a new surface modification option of low cost and naturally abundant halloysite, through in situ photopolymerizations of pyrrole, in order to design reactive and functional bio-based hybrid materials of interest, namely in the humidity sensing field.

## Figures and Tables

**Table 1:** EDX analysis of unmodified and treated HNT samples.

Samples	O	Al	Si	C	Ag	N
HNT	43.4	16.6	14.5	25	0	0
HNT-DMA	46.6	9.5	7.5	34	0	1.7
HNT-DMA-PPy@Ag	45	10.6	8.7	33	0.6	2.7

**Table 2:** Summary of surface chemical composition/Atomic percentage, determined from XPS, for HNT-DMA-PPy@Ag material and precursors. (-) represent the unavailable data or information.

Materials	Si	Al	O	C	N	N(NO_3_)	Ag	Na	K	Ca
HNT	16.5	7.9	62.3	2.10	-	-	-	2.80	0.11	0.50
HNT-NH_2_^*^	24.4	12.0	59.2	9.8	1.50	-	-	0.62	0.67	0.92
HNT-NH_2_-PPy@Ag	14.7	6.7	36.2	38.0	5.28	0.13	2.10	traces	0.30	0.30

* The concentration of the silane is ∼0.05 wt./vol% (weight of aminosilane per 100 mL of solution where HNT is silanized).

**Table 3:** Contact angle measurements of HNT-DMA-PPy@Ag film (0.25 wt%, 0.5 wt%, and 1 wt%).

Sample Type	0.25 w/W% HNT-DMA-PPy@Ag film	0.5 w/W% HNT-DMA-PPy@Ag film	1 w/W% HNT-DMA-PPy@Ag film
Contact angle image	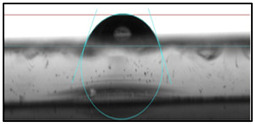	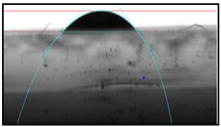	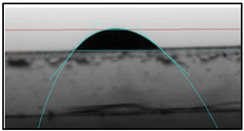
Contact angle	68.2°	46.6°	37°

**Table 4:** Summary of the polypyrrole–NPs based Humidity sensors.

Material	Experimental details	Sensing Range	Response/Recovery time	Reference
Cellulose–PPy nanocomposite	Chemical oxidative polymerization. Time = 30 min at RT CuCl_2_/Py: 10 mL/5 vol% Regenerated cellulose films emerged infiltrate of PPy	30–90%RH	∼418 s	[36]
TiO_2_ NPs/PPy/PMAPTAC)	Photopolymerization Time = 20 min under UV light at RT AgNO_3_/Pyrrole/TiO_2_ NP PMAPTAC/AIBN PET substrate	11–90%RH	30–45 s	[37]
PPy-ZnO nanocomposite	Chemical oxidative polymerization. Time = 60 min at 70 °C PPy-ZnO NP PPy/ZnO: 50 mg/11.8 mg	11–75%RH	180–60 s	[38]
HNT-DMA-PPy@Ag	In situ photopolymerization Time = 20 min at RT HNT/AgNO_3_/Py = 1 g/0.1 M/0.5 M	10–90%RH	30–35 s	This work

## Supplementary Materials

The following are available online at https://www.mdpi.com/2079-4991/10/7/1426/s1, Figure S1: The hysteresis response of the HNT-DMA-PPY-AG ( 0.25 wt%, 0.5 wt%, and 1 wt%) nanocomposite based resistive humidity sensors, Figure S2: Hysteresis response of the HNT-DMA-PPY-Ag ( 0.5 wt%) nanocomposite based humidity sensors.
