# Supplementary material for: Synthesis of In Situ Photoinduced Halloysite-Polypyrrole@Silver Nanocomposite for the Potential Application in Humidity Sensors
Source: Nanomaterials (Basel). 2020 Jul 21;10(7):1426. doi: 10.3390/nano10071426 (PMC7407375; doi:10.3390/nano10071426)

ELECTRONIC SUPPORTING INFORMATION

Synthesis of In Situ Photoinduced Halloysite-Polypyrrole@Silver Nanocomposite for the Potential Application in Humidity Sensors

Khouloud Jlassi [1,*], Shoaib Mallick [2], Hafsa Mutahir [3], Zubair Ahmad [1,*] and Farid Touati [2]

[1] Center for Advanced Materials (CAM), Qatar University, 2713 Doha, Qatar

[2] Department of Electrical Engineering, College of Engineering, Qatar University, 2713 Doha, Qatar; sm1404760@student.qu.edu.qa (S.M.); touatif@qu.edu.qa (F.T.)

[3] Department of Chemical Engineering, College of Engineering, Qatar University, 2713 Doha, Qatar; hm1708761@student.qu.edu.qa

* Correspondence: khouloud.jlassi@qu.edu.qa (K.J.); zubairtarar@qu.edu.qa (Z.A.)

Figure S1: The hysteresis response of the HNT-DMA-PPY-AG(0.25 wt%, 0.5 wt% and 1 wt%) nanocomposite based resistive humidity sensors.

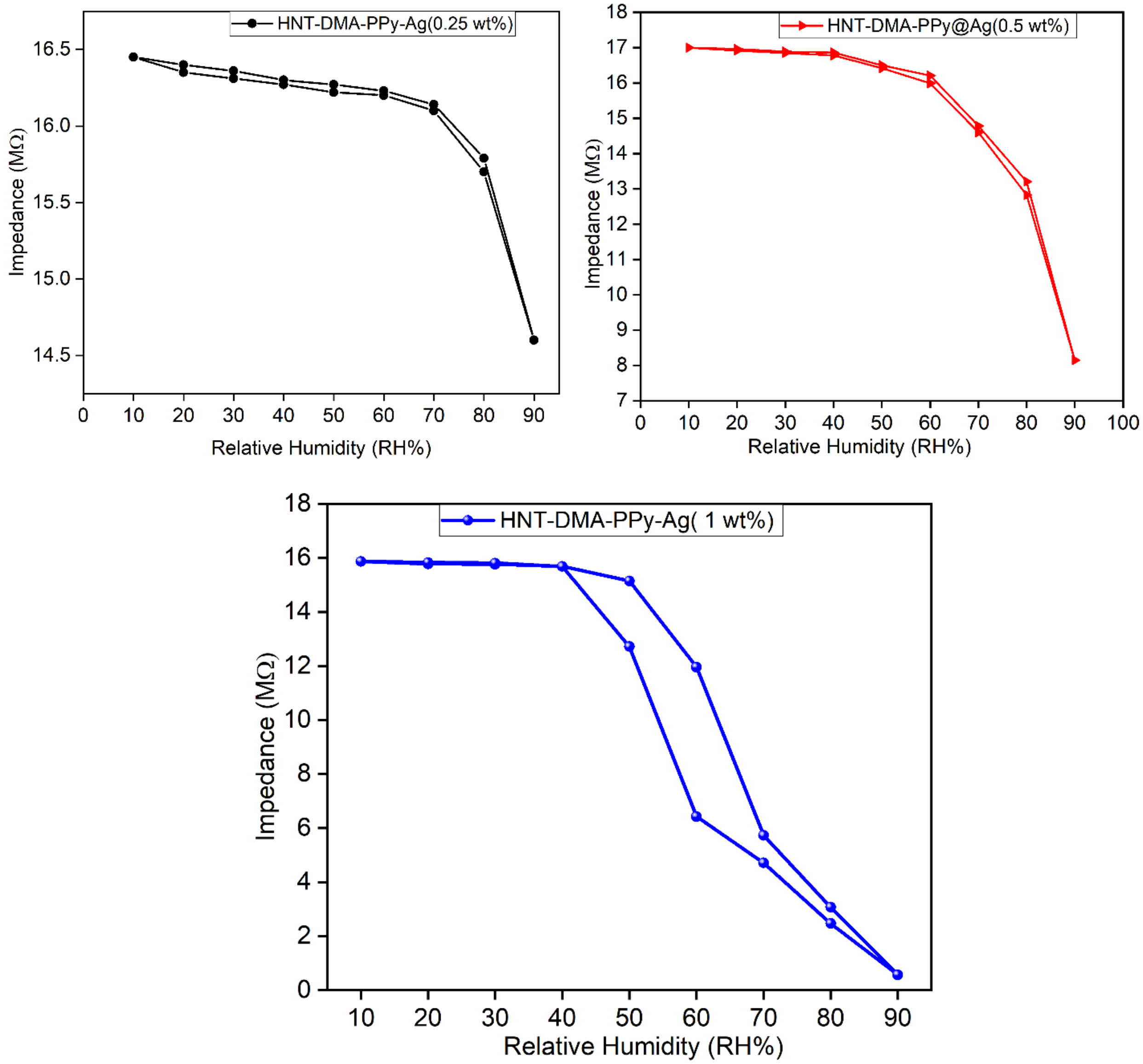

Figure S2: Hysteresis response of the HNT-DMA-PPY-Ag (0.5 wt%) nanocomposite based humidity sensors. Inset shows the response and recovery time(40- 95%RH) of the HNT-DMA-PPY-Ag (0.5 wt%) based impedance sensor.

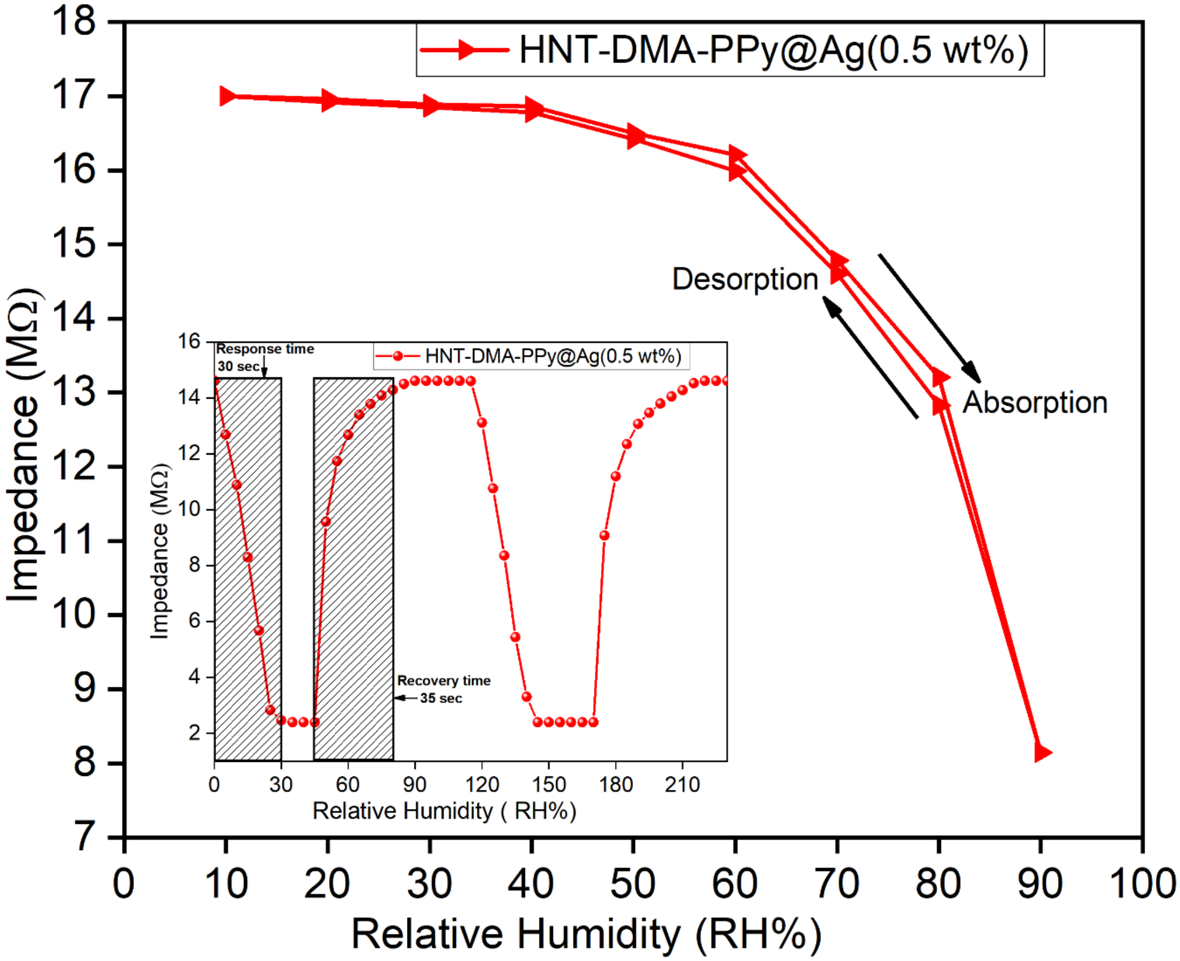

Supplement: Supplementary file 1 [file nanomaterials-10-01426-s001.pdf]
